# Proteome Analysis of Human Natural Killer Cell Derived Extracellular Vesicles for Identification of Anticancer Effectors

**DOI:** 10.3390/molecules25215216

**Published:** 2020-11-09

**Authors:** Jung-Won Choi, Soyeon Lim, Jung Hwa Kang, Sung Hwan Hwang, Ki-Chul Hwang, Sang Woo Kim, Seahyoung Lee

**Affiliations:** 1 Institute for Bio-Medical Convergence, College of Medicine, Catholic Kwandong University, Gangneung-si, Gangwon-do 210-701, Korea; jungwonjian@gmail.com (J.-W.C.); redclover77@hanmail.net (S.L.); kchwang@cku.ac.kr (K.-C.H.); 2 IMMUNISBIO Co. Ltd., B2F MTP Mall, International St. Mary’s Hospital, Incheon Metropolitan City 22711, Korea; kjunghwa@immunisbio.com (J.H.K.); shhwang@immunisbio.com (S.H.H.)

**Keywords:** extracellular vesicles, natural-killer-enriched lymphocytes (NKL), anticancer immunotherapy, proteome analysis

## Abstract

Cancer immunotherapy is a clinically validated therapeutic modality for cancer and has been rapidly advancing in recent years. Adoptive transfer of immune cells such as T cells and natural killer (NK) cells has emerged as a viable method of controlling the immune system against cancer. Recent evidence indicates that even immune-cell-released vesicles such as NK-cell-derived exosomes also exert anticancer effect. Nevertheless, the underlying mechanisms remain elusive. In the present study, the anticancer potential of isolated extracellular vesicles (EVs) from expanded and activated NK-cell-enriched lymphocytes (NKLs) prepared by house-developed protocol was evaluated both in vitro and in vivo. Moreover, isolated EVs were characterized by using two-dimensional electrophoresis (2-DE)-based proteome and network analysis, and functional study using identified factors was performed. Our data indicated that the EVs from expanded and active NKLs had anticancer properties, and a number of molecules, such as Fas ligand, TRAIL, NKG2D, β-actin, and fibrinogen, were identified as effector candidates based on the proteome analysis and functional study. The results of the present study suggest the possibility of NK-cell-derived EVs as a viable immunotherapeutic strategy for cancer.

## 1. Introduction

Cancer immunotherapy designed to boost the immune system to produce antitumor effects has been scrutinized for its clinical viability [1]. Despite the disappointing results of early trials, immunotherapy is now rapidly advancing and being a clinically proven treatment modality for various types of cancers [2]. Immunotherapeutic strategies include, but are not limited to, cancer vaccines, oncolytic viruses, adoptive cell transfer, and administration of antibodies or recombinant proteins [2]. Especially, the adoptive transfer of immune cells such as T cells and natural killer (NK) cells has emerged as a targeted method of controlling the immune system against cancer [3].

NK cells, as a member of the lymphocytes of the innate immune system, have cytotoxic ability against both virus-infected cells and tumor cells [4]. Because of their ability to recognize and kill tumor cells without antigen exposure, they have been considered promising agents for cell-based cancer therapies [4]. However, the scarcity of NK cells in human lymphocytes, their changing phenotype, and their impaired functionality during cancer progression necessitates the development of protocols to activate and expand NK cells to sufficient numbers ex vivo for adoptive transfer [4,5]. We have developed a feeder-free NK cell expansion system where clinically viable NK cells are isolated and cultured from human peripheral blood mononuclear cells (PBMCs) for NK studies [6]. 

Another approach to bypass the current limitations of NK-cell-based immunotherapy is the use of NK-cell-derived extracellular vesicles (EVs) without using actual NK cells. EVs are found in various body fluids and originate directly from the plasma membrane of the cell [7]. They participate in intercellular communication by transporting mRNA, miRNA, and proteins [8,9] and have various functions depending on the cell types they are derived from [10]. In the tumor microenvironment, all cells, including endothelial cells, pericytes, and immune cells, as well as tumor cells, release EVs [11]. While tumor-cell-derived EVs mediate angiogenesis, invasion, immune escape, chemotherapy resistance, and phenotypic modification of recipient cells [12], immune-cell-derived EVs suppress tumor progression by direct or indirect mechanisms [11]. Such effects of immune-cell-derived EVs enable one to create new methods for cancer therapy. However, the heterogeneity of immune-cell-derived EVs and their unclarified mechanisms prevent them from being an effective therapeutic modality against cancer. Therefore, the characterization of immune-cell-derived EVs would be the first step for assessing their therapeutic potential against cancers.

Immune-cell-derived EVs are reported to have anticancer properties [11,13]. In the present study, we examined the anticancer potential of EVs isolated from human NK-cell-enriched lymphocytes (NKLs) prepared by a house-developed protocol. EVs can be classified based on their sizes as exosomes (30–100 nm), microvesicles (100–1000 nm), and apoptotic bodies (>1000 nm) [14]. Exosomes are generated through fusion with the plasma membrane of specific endosomal compartments [15], and microvesicles are produced via an outward budding from the plasma membrane [16]. Although their cargo, membrane composition, and surface molecules are distinct with some overlapping features, exosomes and microvesicles often share functions, and their sizes can be similar [14].

In the present study, we characterized EVs isolated from expanded and activated NKL using two-dimensional electrophoresis (2-DE)-based proteome analysis and evaluated their therapeutic potential against cancer both in vitro and in vivo to identify potent anticancer effectors of NKL-derived EVs that may help to develop an anticancer cocktail to treat cancer even without using the original EVs.

## 2. Results

### 2.1. Preparation of Ex Vivo Expanded NKLs

**Figure 1:**
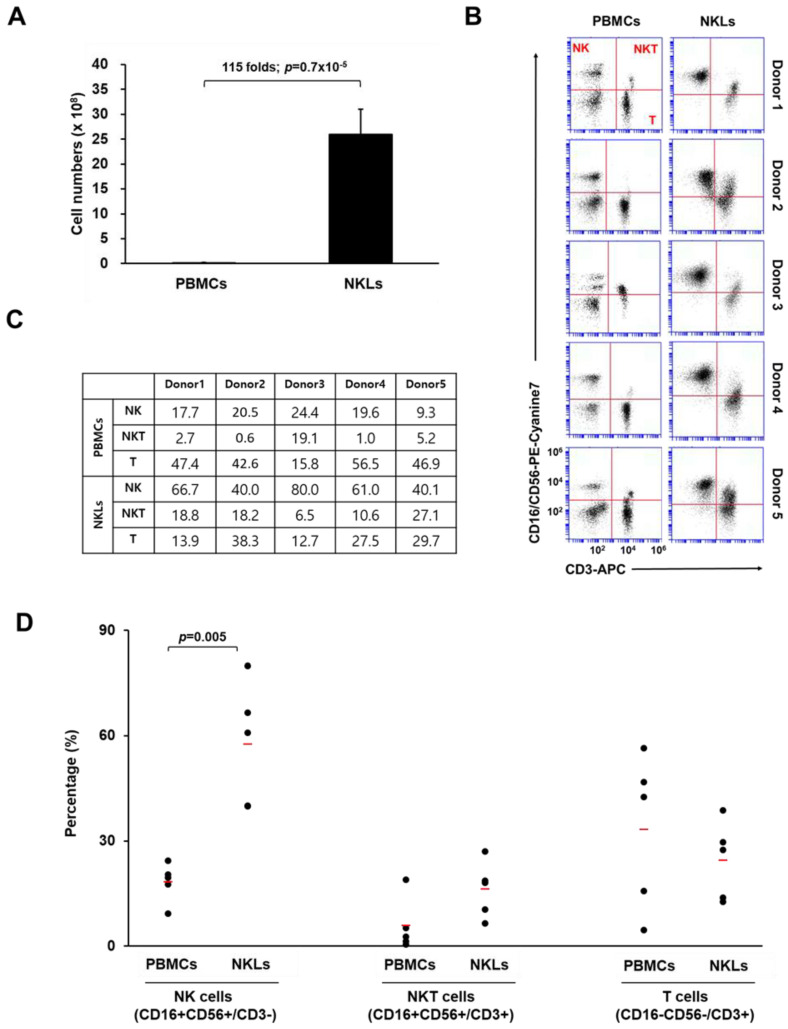
Characterization of expanded natural killer (NK)-cell-enriched lymphocytes (NKLs). (**A**) Comparison of total cell numbers. (**B**) Distribution of NK (CD3−CD16+CD56+), NKT (CD3+CD16+CD56+), and T cells (CD3+CD16−CD56−) compared between peripheral blood mononuclear cells (PBMCs) and NKLs from 5 individuals. (**C**) Percentages of NK, NKT, and T cells before and after the in vitro expansion. (**D**) The mean percentage of each group is indicated with red bars. Significant differences between PBMCs and NKLs were determined via analysis of variance (ANOVA).

To expand NKLs from PBMCs, blood-isolated PBMCs were cultured in the presence of agonistic antibodies against activating receptors (CD16 and CD56) and a natural cytotoxic receptor (NKp46) of NK cells, selected cytokines (IL-2, IL-12, and IL-18), and autologous human plasma. After 2 weeks of culture, the total cell number of expanded NKLs increased approximately 115-fold compared to that of initially isolated PBMCs (2.1 × 10^7^ vs. 2.5 × 10^9^ cells, Figure 1A). The proportion of NK (CD16+CD56+/CD3−), NKT (natural killer T cell, CD16+CD56+/CD3+), and T cells (CD16−CD56−/CD3+) in initially isolated PBMCs and NKLs was determined using flow cytometry. In the initially isolated PBMCs, the proportions of NK cells and NKT cells were 18.3 ± 5.6% and 5.8 ± 7.6%, respectively. After 2 weeks of cultivation, these proportions increased to 57.6 ± 17.4% and 16.2 ± 8%, respectively, in NKLs (Figure 1B,C). By contrast, the percentage of T cells in NKLs slightly decreased compared to PBMCs (Figure 1D), indicating that NK/NKT cells were preferentially expanded compared to T cells under the given culture conditions. We used a CD16/CD56 cocktail for this particular flow cytometry analysis due to a limited multicolor processing capacity of the device, as we previously found that the expression patterns of CD3/CD56-stained PBMCs and CD3/CD16-stained PBMCs were highly identical (data not shown). Nevertheless, the possibility of mixed-up presentation of CD56dimCD16+ cells and CD56brightCD16− cells, as well as of just-activated T cells being recognized as NKT cells, exists. This remains one of the limitations of this study and should be improved for further studies.

### 2.2. Isolation of EVs from Culture Medium of NKLs

**Figure 2:**
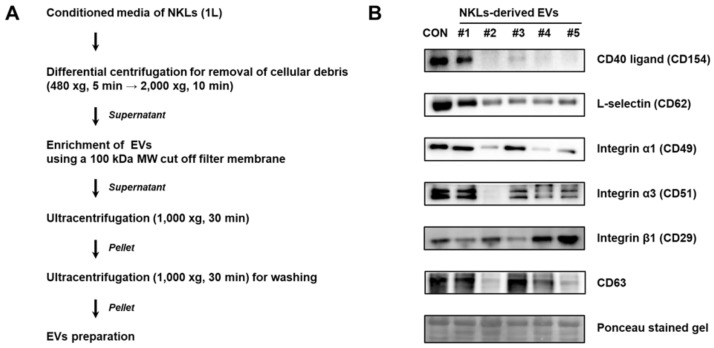
(**A**) Experimental scheme for extracellular vesicle (EV) isolation from conditioned medium of NKLs (**B**) Expression of markers in isolated EVs. Control EVs (CON) were prepared from concentrated medium containing pooled autologous plasma of 5 individuals.

EVs were isolated from the culture medium of NKLs following the protocol shown in Figure 2A. Primary verification of isolated EVs was done by immunoblot analysis using EV markers such as selectins, integrins, and CD40 known to be contained in EVs [9]. CD40 ligand (CD154) was detectable in control, EV 1, and EV 3. For integrin α5 (CD49a), all the samples except EV 2 expressed CD49a. For all other markers examined, although there was interindividual difference, most of the NKL-derived EVs and control EVs expressed examined markers, namely L-selectin (CD62L), integrin α1 (CD49a), integrin β1 (CD29), and CD63 tetraspanin, a common exosome marker [17] (Figure 2B).

### 2.3. Anticancer Effect of NKL-Derived EVs In Vitro and In Vivo

**Figure 3:**
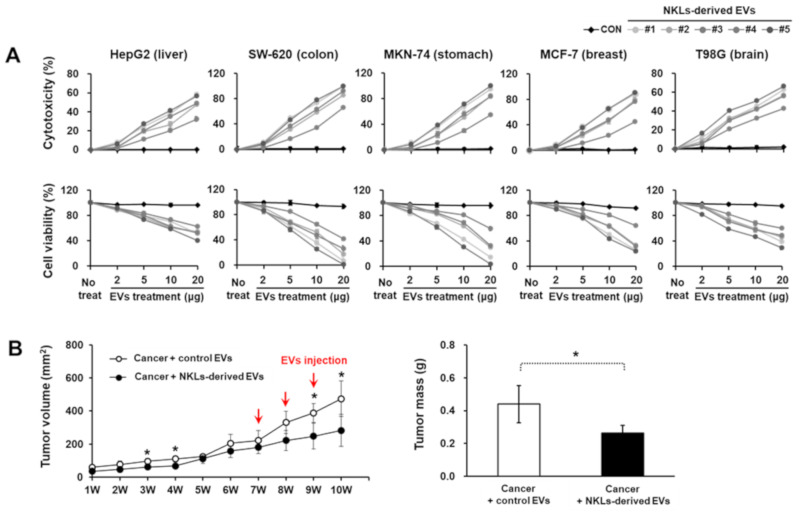
Cytotoxic potential of isolated EVs against cancer cell lines and cancer animal model. (**A**) Isolated individual EVs were used to treat 5 cancer cell lines from different tissues; cytotoxicity of isolated EVs against cancer cells was observed, and cell viability of cancer cells against isolated EVs was measured. Experiments were performed in triplicate. Significant differences between untreated cancer cells and EV-treated cancer cells were determined via ANOVA. (**B**) The volume of tumor and tumor mass after administration of vehicle (PBS) and NKL-derived EVs in MCF-7-cell-injected female athymic nude mice. The tumor volume was monitored every week for 10 weeks (*n* = 4 for each group), and tumor mass was measured at the end of the animal study. Significant differences between groups were determined via ANOVA, with *p*-values indicated as * *p* < 0.05.

To evaluate the anticancer potential of NKL-derived EVs in vitro, EVs at four different concentrations (2, 5, 10, and 20 μg/well) were applied to five different cancer cell lines with different origins (HepG2, liver; SW-620, colon; MKN-74, stomach; MCF-7, breast; T98G, brain). As the concentration of EVs increased, the cell viability of all five cancer cell lines significantly decreased, while lactate dehydrogenase (LDH) release significantly increased (Figure 3A). Furthermore, in in vivo study using an MCF-7-based breast cancer model, NKL-derived EVs started to significantly suppress the tumor growth from 2 weeks after the injection and thereafter (388.3 ± 58 mm^3^ vs. 248.5 ± 78.4 mm^3^ in control group and NKL-derived EV group, respectively) (Figure 3B). The final tumor mass was significantly smaller in the NKL-derived EV group (0.27 ± 0.05 g) than in the control group (0.44 ± 0.11 g) as well (Figure 3B). Since no healthy, normal cells were analyzed in the cytotoxicity assays to exclude an unspecific cytotoxic effect of the NKL-derived EVs in the present study, there can be concern. However, regarding possible cytotoxicity of EVs on healthy normal cells, we have examined the cytotoxicity of NKL-derived EVs prepared by using the same protocol on human adipose-derived stem cells (ASCs) in a different study. According to our unpublished data, the NKL-derived EVs had no significant cytotoxic effect on human ASCs for up to 48 h of co-culture, and therefore, it was assumed that there is no significant cytotoxicity of the NKL-derived EVs against healthy, normal cells.

### 2.4. Expression of Anticancer-Activity-Related Proteins in NKL-Derived EVs

**Figure 4:**
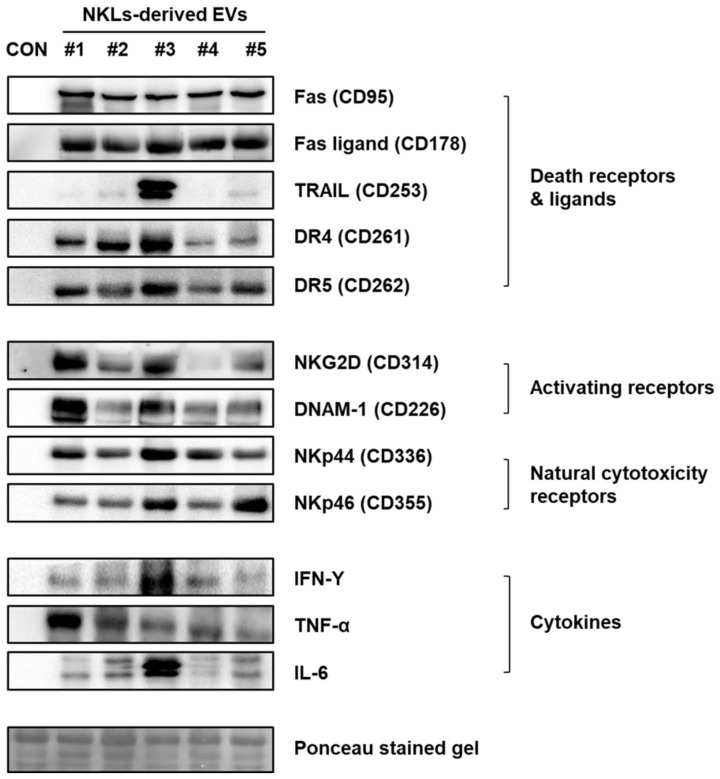
Expression of receptors, ligands, and cytokines related to cytotoxicity against cancer cells in NKL-derived EVs. CON: control EVs.

To identify the effector proteins presumably account for the observed anticancer effect of NKL-derived EVs in vitro and in vivo, the expression of well-known anticancer mediators, such as death receptors (Fas/APO-1/CD95, DR4/CD261/TRAILR1, and DR5/CD262/TRAILR2) and ligands (Fas ligand/CD178 and TRAIL), activating receptors (NKG2D/CD314 and DNAM-1/CD226), natural cytotoxicity receptors (NKp44/CD336 and NKp46/CD355), and cytokines (IFN-γ, TNF-α, and IL-6) was examined by using immunoblot analysis (Figure 4). Although there was individual variation among five donors, the expression of death receptors and ligands, activating and natural cytotoxicity receptors, and cytokines was prominent in the NKL-derived EVs, while none of them were detected in control EVs (Figure 4).

### 2.5. Separation and Identification of Significantly Increased EV Proteins Using Proteome Analysis

**Figure 5:**
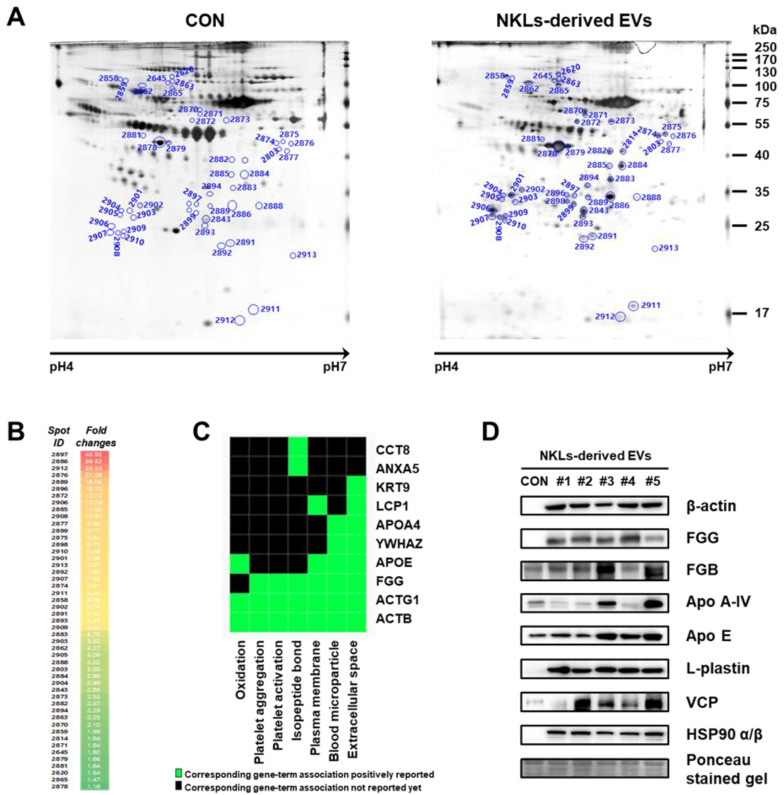
Proteome analysis of NKL-derived EVs. (**A**) Representative silver-stained two-dimensional electrophoresis (2-DE) gel images of control EVs (CON) and NKL-derived EVs (**B**) Spots with increased intensity and their fold changes in NKL-derived EVs compared to CON. A high-resolution image along with 2-DE gel images is presented in Figure S1. (**C**) Gene-Term 2D Heat map view using DAVID Bioinformatics resources. (**D**) The expression of increased proteins was confirmed by immunoblot analysis. 2-DE experiments were performed in triplicate per individual.

To find other novel proteins that might have facilitated the tumor-killing of NKL-derived EVs, 2-DE-based proteome analysis was performed using isolated EVs. EV proteins were separated by 2-DE, and nearly 630 individual spots (mass ranging from 6 to 240 kDa and pH between 4 and 7) were detected (Figure 5A). Among them, a total of 49 spots significantly increased in the NKL-derived EV, and 37 of them were identified by peptide mass fingerprinting (PMF) (Figure 5B (also see Figure S1 for a high-resolution image) and Table 1). It was interesting to notice that 5 spots (out of 27) were different fibrinogen isotypes and 12 spots were β-actin fragments (and there was an additional 1 γ-actin fragment) (Table 1). For functional classification of the identified proteins, a Gene-Term 2D Heat map was constructed using DAVID (https://david.ncifcrf.gov/) (Figure 5C). Most of the identified proteins could be categorized under the annotation terms of extracellular space, blood microparticle, and plasma membrane based on the enrichment scores. Additional immunoblot analysis to exclude the possibility of technical errors and artificial effects during proteome analysis was performed for eight selected proteins of interest, namely β-actin, FGG, FGB, Apo A-IV, Apo E, L-plastin, VCP, and HSP90 α/β, and the results of the immunoblot analysis also confirmed that those proteins were highly expressed in NKL-derived EVs compared to the control (Figure 5D).

### 2.6. Functional Verification of the NKL-Derived EV Proteins Using Neutralizing Antibodies

**Figure 6:**
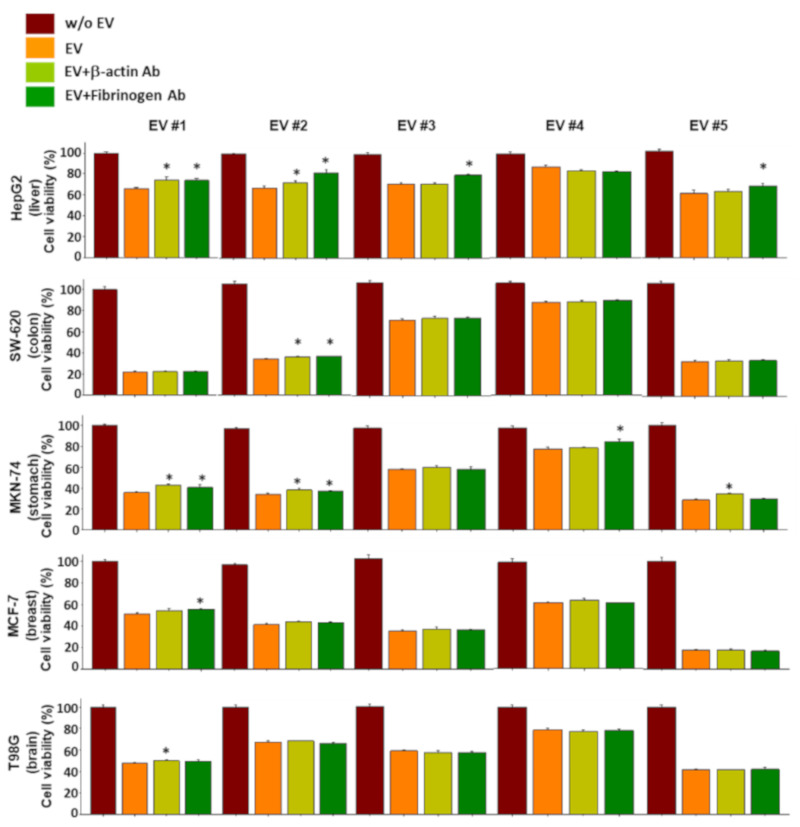
Effect of neutralizing antibodies against β-actin and fibrinogen on the anticancer effect of EVs. Individual EVs mixed with/without neutralizing antibodies specific to β-actin and fibrinogen were applied to 5 different types of cancer cells, and viability of cancer cells was measured. Experiments were performed in triplicate. Significant differences were determined via ANOVA, with *p*-values indicated as * *p* < 0.05 compared to the EVs without neutralizing antibodies.

**Figure 7:**
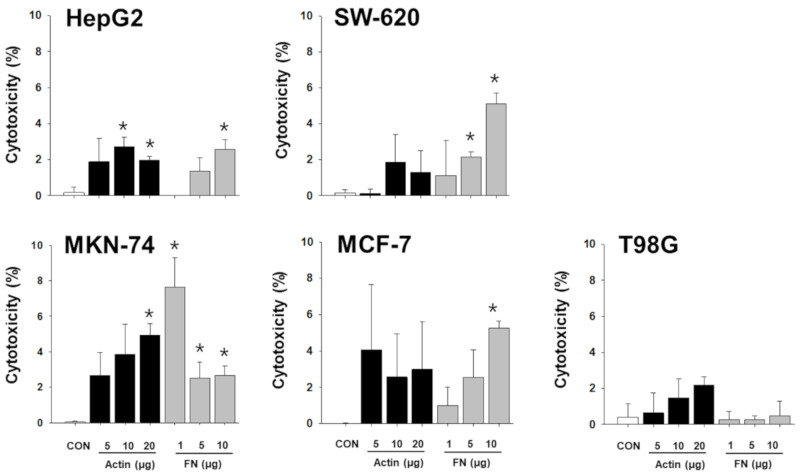
Cytotoxic effect of recombinant β-actin and fibrinogen on different types of cancer cells. Varying concentrations of recombinant β-actin and fibrinogen were applied to 5 different types of cancer cells, and cytotoxicity on cancer cells was measured. Experiments were performed in triplicate. Significant differences were determined via ANOVA, with *p*-values indicated as * *p* < 0.05 compared to untreated control (CON). Actin: β-actin, FN: fibrinogen.

To verify whether the proteins of the NKL-derived EV actually contributed to the observed anticancer effect, a functional study using neutralizing antibodies was conducted. Although the results were inconsistent and showed an intraindividual variability, the proteins examined contributed to the observed anticancer effect of NKL-derived EVs to a certain extent (Figure S2). Interestingly, even the neutralizing antibodies against β-actin and fibrinogen negated the anticancer effect of EVs, especially in liver cancer cells and stomach cancer cells (Figure 6). Furthermore, treatment with recombinant β-actin and fibrinogen showed marginal, but statistically significant, anticancer effect in certain types of cancer cells (Figure 7), suggesting that β-actin and fibrinogen actually contributed to the observed anticancer effect of the NKL-derived EVs. On the other hand, when the data were analyzed as pooled data, all the significance observed was completely masked (data not shown), and this also indicated that the observed effects in single patients were donor-specific.

## 3. Discussion

In the present study, the isolated EVs were found to express commonly known microvesicle markers such as selectins, integrins and the CD40 ligand [9], and exosome marker CD63 expression was also detected (Figure 2B). The CD63 is a member of the tetraspanin superfamily of integral membrane proteins and is known as an exosome marker along with CD9 and CD81 [17], but it was first known as a marker of platelet activation [18]. Since the EV isolation protocol used in the present study did not have a platelet depletion step (Figure 2A) and CD63 is not only enriched in the platelet-derived exosomes but also present on platelet-derived microvesicles [19], it was assumed that the isolated EVs were mostly microvesicles rather than a mixture of microvesicles and exosomes. 

Since our in vitro expansion protocol involves culturing PBMCs in the presence of donor’s plasma, which also may contain EVs, concerns can be raised about possible contamination of samples with EVs from donor’s plasma. This issue is closely linked to the half-life of EVs (including both exosomes and microvesicles), and a previous study examining the half-life of exosomes indicated that exosomes have an average half-life of 4 min and approximately 10% of them remain intact after 4 h [20]. Furthermore, microvesicles may have an even shorter half-life because their membrane lipids can be hydrolyzed by blood-borne phospholipases [21,22]. Considering that our protocol to produce NKLs uses 0.5% human plasma at the final step and the cells are cultured for additional 6–7 days before harvesting, contamination of the sample by EVs from the donor’s plasma is less likely.

To date, a variety of immune-cell-derived EVs has been reported to suppress tumor progression [11]. Dendritic cell (DC)-derived EVs with major histocompatibility complex (MHC) class I and class II peptide complexes were able to prime other immune cells and activate an antitumor immune response [23]. Phase I clinical trials have demonstrated the safety of using DC-derived exosomes in patients with metastatic melanoma [24] and lung cancer [25], and phase II clinical trials have shown that DC-derived exosomes can increase NK cell functions in non-small-cell carcinoma patients [26]. The combination of exosomes and the invariant NKT immune cell ligand α-galactosylceramide (αGC) induced potent NK and γδ T cell innate immune responses in vitro and in vivo, and they increased survival by increasing antigen-specific CD8+ T cell tumor infiltration while decreasing tumor growth in an ovalbumin (OVA)-expressing tumor-bearing mouse model [27]. According to a recent study, NK92-cell-derived exosome also exerts antitumor effects against aggressive melanoma in vitro and in vivo, while not showing any significant cytotoxicity against healthy, normal cells [28]. Therefore, it seems that NK-cell-derived exosomes can preferentially kill tumor cells over normal, healthy cells, suggesting that NK-cell-derived exosomes can discriminate between tumor cells and non-tumor cells [29] and that the possibility of NKL-derived EVs having cytotoxicity against normal, healthy cells is very small. Nevertheless, the underlying mechanisms of EVs derived from immune cells remain largely unknown, and the identification of anticancer effector molecules contained in the EVs might be the first step to comprehend the mechanism.

To find effector molecules responsible for the observed in vitro and in vivo anticancer effect of NKL-derived EVs, some of the well-known candidate molecules were examined first. NK cells express activating (NKG2D and DNAM-1) and inhibitory receptors (KIR and CD94) and natural cytotoxicity receptors (NKp30, NKp44, and NKp46). These receptors recognize MHC class I and related molecules and cellular ligands, and they are able to induce or block NK cell responses [30]. The cytotoxicity of NK cells is modulated by the balance between activating and inhibitory signals mediated by the receptors expressed on the cell surface [31]. Additionally, NK cells also express death receptors (Fas, DR4, and DR5) and ligands (FasL and TRAIL) [32], and TRAIL-DR4/5-mediated cytotoxicity plays an important role in eliminating the target cells [33]. As to the important soluble factors, NK cells produce IFN-γ, which inhibits viral replication directly, and secrete pro-inflammatory cytokines such as TNF-α and IL-6 along with various growth factors and chemokines [34]. According to the immunoblot data, the isolated EVs also contain these well-known anticancer mediators despite obvious interindividual variation in expression (Figure 4), suggesting that these factors might have also mediated the observed anticancer effect of the NKL-derived EVs as well.

For the identification of additional effector molecules that might have modulated the observed anticancer effect of the NKL-derived EVs, 2-DE-based proteome analysis was conducted, and it identified 37 different candidate proteins out of 49 proteins significantly increased in the NKL-derived EVs. Some of them are known to influence the function of NK and/or T cells, but the majority of them have no known relationship with NK and/or T cells (Table 1 and Figure 5). 

For example, L-plastin, a family of actin-binding proteins, is one of the 37 proteins identified in the present study, and it was reported that l-plastin facilitates NKG2D-mediated NK cell migration [35] and T cell activation [36]. HSP90, another protein found to be increased in the NKL-derived EVs, is important for the regulation of phenotype and functional activity of human T lymphocytes and NK cells [37]. On the other hand, it was intriguing to notice that out of 37 increased spots, 5 spots were fibrinogen isotypes and 13 spots were β/γ-actin fragments (Table 1); these fibrinogen isotypes and β/γ-actin fragments also contributed to the observed anticancer effect of the NKL-derived EVs under certain circumstances, as evidenced by the antibody-mediated blocking experiments (Figure 6 and Figure S2). To be exact, although the effect was marginal, β-actin-neutralizing antibodies significantly attenuated the anticancer effect of the EV 1 in more than three different types of cancer cells (HepG2, MKN-74, and T98G) (Figure 6), and fibrinogen-neutralizing antibodies attenuated the anticancer effect of the EVs 1, 3, and 5 in HepG2 and that of the EVs 1, 2, and 4 in MKN-74 (Figure 6). This anticancer effect of β-actin and fibrinogen was further confirmed by the experiment using recombinant β-actin and fibrinogen without EVs (Figure 7).

These findings are surprising because β-actin and fibrinogen are the molecules least expected to contribute to the observed anticancer effect of the NKL-derived EVs. For example, the actin family is a family of globular multifunctional proteins that play roles in many critical cellular processes, including muscle contraction, cell motility, cell division, vesicle, cell signaling, and the establishment and maintenance of cell junctions and cell shape [38]. Regarding cancers, abnormal expression and/or polymerization of the actin cytoskeleton profoundly affect the invasiveness and metastasis of cancers [39]. More relevant to the present study, actin cytoskeleton rearrangement occurs during clathrin-mediated endocytosis [40], one of the major mechanisms by which EVs enter target cells [41]. Therefore, it may be feasible that the actin-neutralizing antibodies drawn into cancer cells during the first round of endocytosis bind to the actin filaments and thus hinder endocytosis. Nevertheless, this has to remain speculation without further empirical evidence to support it at this point. In addition, the present study did not cover the mechanism behind the increase of these unlikely molecules in the first place, nor did it identify the exact location of those molecules (i.e., bound to the surface of EVs or wrapped up inside). To answer all these unanswered questions, especially the important question of how those molecules contribute to the anticancer effect of the NKL-derived EVs, further studies are warranted.

The goal of the present study was not to test the feasibility of using the NKL-derived EVs for directly treating cancers; instead, it aimed to find any novel anticancer effectors of the NKL-derived EVs, as the identification of such effectors could make it possible to develop an anticancer cocktail to treat cancer even without using the original EVs. Furthermore, doing so could bypass the issues such as high heterogeneity or low reproducibility of the original EVs.

## 4. Materials and Methods 

### 4.1. Donors

Human PBMCs and plasma used for the experiment were obtained from human blood samples of 5 healthy donors recruited at the International St. Mary’s Hospital of Catholic Kwandong University. All donors provided informed consent to participate, and the study protocol was approved by the Institutional Review Board of the International St. Mary’s Hospital, Catholic Kwandong University (IS18TSSE0043).

### 4.2. Isolation of PBMCs/Plasma and Preparation of NKLs

A house protocol describing the preparation of ex vivo expanded NKLs has been recently published [6]. Briefly, blood samples were collected from healthy donors, and PBMCs and plasma were collected from buffy coats and the upper aqueous phase of blood, respectively, by density gravity centrifugation (Ficoll-Paque, GE Healthcare, Piscataway, NJ, USA). For expansion and activation of NKLs, isolated PBMCs were supplemented with the following bioactive components: 0.5% or 10% autologous human plasma depending on the step, 3 different agonistic antibodies, and 3 different cytokines. The cells were cultured for 2 weeks to produce a sufficient number of NKLs. The following antibodies and cytokines were used: anti-human CD56 (555513, BD Biosciences, San Jose, CA, USA), anti-human CD16 (555403, BD Biosciences), anti-human CD355 (MAB1850-500, R&D SYSTEMS, Minneapolis, MN, USA), IL-2 (653601261, Novartis, Whippany, NJ, USA), IL-12 (200-12, PeproTech, Rocky Hill, NJ, USA), and IL-18 (B003-2, R&D Systems).

### 4.3. Flow Cytometry

To detect NK, NKT, and T cells using specific surface markers, NKLs were incubated with flow cytometry staining buffer for 30 min on ice and then washed twice with PBS. The staining buffer was a buffered saline solution containing fetal bovine serum (FBS, 26140079, Gibco by Life Technologies, Grand Island, NY, USA), 0.09% sodium azide (S2002, Sigma-Aldrich, St. Louis, MO, USA) anti-human CD56 PE-Cyanine7 (25-0567-42, eBioscience, San Diego, CA, USA), anti-human CD16 PE-Cyanine7 (25-0168-42, eBioscience), and anti-human CD3 APC (17-0036-42, eBioscience) at a final concentration of 0.125 μg. The stained samples were analyzed and data were processed on an Acurri6 flow cytometer (BD Biosciences). The PBMCs were analyzed immediately after collecting blood from donors, whereas NKLs were examined by flow cytometry after two weeks of culturing using house-developed protocol. Though two experiments were performed on different days, we used the same flow cytometry conditions such as compensation, threshold, gating, and cell numbers to minimize possible technical variations between the two experiments.

### 4.4. Isolation of EVs from Culture Medium of NKLs

Culture medium of NKLs (1 L) and the same amount of fresh medium devoid of cells but containing an equal amount of autologous pooled plasma from 5 healthy donors were centrifuged at 480× *g* for 5 min and 2000× *g* for 10 min to remove intact cells and cell debris [42]. The resulting supernatant was concentrated to 25 mL using a 100 kDa MW cut-off filter membrane (PN OA100C12, Minimate TFF capsule, Pall Corporation, Ann Arbor, MI, USA). The concentrated conditioned media (CM) and control sample were further ultracentrifuged at 10,000× *g* for 30 min twice to obtain NKL-derived EVs and control EVs (Figure 2A). Isolated EVs were stored at −70 °C until use.

### 4.5. Immunoblot Analysis

The expression of known EV markers, cytokines, receptors, and ligands was examined by immunoblot analysis according to the methods previously published [43]. Briefly, EV lysates were prepared with RIPA buffer (89901, Thermo Scientific, Rockford, IL, USA) containing protease inhibitor and phosphatase inhibitor, and 40 μg of EV lysates per lane was used for SDS–polyacrylamide gel electrophoresis (PAGE). The proteins were transferred to polyvinylidene fluoride (PVDF, Santa Cruz Biotechnology, Santa Cruz, CA, USA) membranes. The membranes were then incubated with appropriate primary antibodies and horseradish peroxidase (HRP)-conjugated secondary antibodies (Santa Cruz Biotechnology). Immunolabeled bands were visualized using an enhanced chemiluminescence (RPN2232, GE Healthcare) system. The following antibodies (purchased from Santa Cruz Biotechnology, Dallas, TX, USA) were used for the study: anti-CD154 (sc-74448), anti-l-selectin (sc-7297), anti-integrin α1 (sc-271034), anti-integrin α3 (sc-374242), anti-integrin β1 (sc-374429), anti-CD63 (sc-365604), anti-FAS (sc-8009), anti-FAS ligand (sc-33716), anti-TRAIL (sc-8440), anti-DR4 (sc-8411), anti-DR5 (sc-166624), anti-NKG2D (sc-53501), anti-DNAM-1 (sc-376736), anti-NKp44 (sc-59342), anti-NKp46 (sc-53599), anti-IFN-γ (sc-373727), anti-TNF α (sc-52746), anti-IL-6 (sc-28343), anti-β-actin (sc-517582), anti-FGG (sc-133226), anti-FGB (sc-271035), anti-ApoA-IV (sc-374543), anti-ApoE (sc-13521), anti-l-plastin (sc-133218), anti-VCP (sc-57492), and anti-HSP90 α/β (sc-13119).

### 4.6. Culture of Cancer Cell Lines

HepG2 (hepatoblastoma, HB-8065, ATCC, Manassas, VA, USA), SW-620 (colorectal carcinoma, CCL-227, ATCC), MKN-74 (stomach adenocarcinoma, 80104, Korea Cell Line Bank, Seoul, Korea), MCF-7 (breast adenocarcinoma, HTB-22, ATCC) and T98G (brain glioblastoma, CRL-1690, ATCC) cells were used. The SW-620, MKN-74, and MCF-7 cells were cultured in RPMI 1640 Medium (21870076, Gibco by Life Technologies) and the HepG2 and T98G cells were cultured in Minimum Essential Medium (MEM, 11090073, Gibco by Life Technologies) supplemented with 10% heat-inactivated FBS, 2 mM L-glutamine (25030081, Gibco by Life Technologies), 1 mM sodium pyruvate (11360070, Gibco by Life Technologies), and 25 mM HEPES (15630080, Gibco by Life Technologies). The cells were maintained in a humidified atmosphere of 5% CO2 and 95% air at 37 °C.

### 4.7. Cell Viability and Cytotoxicity Assay

Five different cancer cell lines (HepG2, SW-620, MKN-74, MCF-7, and T98G) were seeded at a density of 1 × 10^4^ cells/well in a 96-well plate. After 24 h, isolated EVs from CM of NKL were applied to the five cancer cells with different concentrations (2, 5, 10, and 20 μg/well). After 24 h of culture, the supernatants were collected and objected to LDH assay to determine the cytotoxicity of EVs against cancer cells using a cytotoxicity detection kit (MK401, Takara, Nojihigashi, Kusatsu, Shiga, Japan). The viability of EV-treated cancer cells was measured using Ez-Cytox (Ex-3000, DOGEN, Seoul, Korea).

### 4.8. Xenograft Model Using MCF-7

Female athymic nude mice (5 weeks, Koatech, Pyeoungtaek, Korea) were used for the in vivo study. To evaluate the anticancer efficacy of the EVs isolated from CM of NKLs, the animals were subcutaneously bolus injected with MCF-7 (3 × 10^6^ cells/head) in the right flank. To facilitate breast tumor growth, the animals were also received 17β-estradiol valerate (1 µg in 100 μL of peanut oil/head, E2758, Sigma-Aldrich) every three days starting from three days prior to the MCF-7 injection. Tumor volume (width × width × length/2) reached approximately 200 mm^3^ at week 7, and the animals were randomly assigned to control EV group (*n* = 4) and NKL-derived EV group (*n* = 4). These two groups received control EVs and NKL-derived EVs (50 μg in 100 μL of PBS), respectively, via tail vein once a week for 3 weeks. Control EVs were concentrated EVs with an equal amount of autologous pooled plasma from 5 donors. The tumor volume was measured once per week after MCF-7 cell injection. At the end of the in vivo study (10 weeks after the MCF-7 injection), the animals were sacrificed, and excised tumor weight was measured. All experimental procedures for animal studies were approved by the Committee for the Care and Use of Laboratory Animals of Catholic Kwandong University College of Medicine and were performed in accordance with the Committee’s Guidelines and Regulations for Animal Care (CKU 01-2017-008).

### 4.9. Preparation of Protein Samples for 2-DE

For 2-DE analysis, proteins were isolated from the phenol–ethanol supernatant layer left over after the DNA precipitation step using TRIzol reagent (15596026, Thermo Fisher, Waltham, MA, USA). Precipitated proteins were dissolved in a rehydration buffer containing 7 M urea, 2 M thiourea, 4% CHAPS, 20 mM DTT, 1 mM PMSF, 2% immobilized pH gradient (IPG) buffer (Ampholyte 3/10, Bio-Rad, Hercules, CA, USA), and a trace of bromophenol blue. The proteins were then stored at −80 °C until analysis. Protein content was determined using Bradford protein assay (23236, Thermo Fisher).

### 4.10. 2-DE Analysis

2-DE was performed in triplicate using EV protein samples (150 µg per gel) from 5 healthy donors. IPG isoelectric focusing (IEF) of protein samples was performed at pH 4–7 with 18-cm IPG DryStrips (17-1233-01, GE Healthcare) using the Ettan IPGphor 3 system (GE Healthcare) according to the protocol recommended by the manufacturer. The IPG strips were passively rehydrated for 12 h in strip holders with 340 µL of DeStreak Rehydration Solution (17600319, GE Healthcare), which contained 30 µg of protein in each sample. IEF was executed using the advanced mode protocol: 1 h at 500 V, 3 h at 1000 V, 6 h at 7000 V, and finally at 7000 V until it reached 115 KVh. The gel strips were then placed onto a 12% polyacrylamide gel for resolving along the second dimension, using an Ettan DALTsix system. A total of 18 gels (3 gels from control and 15 gels from five individual samples) were visualized using silver staining and submitted to image analysis and peptide mass fingerprinting (PMF) [44].

### 4.11. Image Acquisition and Data Analysis

The gels were imaged using a UMAX PowerLook 1120 system (UMAX Technologies, Inc., Dallas, TX, USA, and modified ImageMaster 2-D software V4.95 (GE Healthcare) was used to compare the images. The detected spots from all gels were matched with those in the reference gel, which was selected from the control gels. Relative optical densities and relative volumes were calculated to correct differences in in-gel staining. The intensity volume of each spot was calculated using background subtraction and total spot volume normalization, and the resulting spot volume percentage was used for group comparison.

### 4.12. Protein Identification

Protein spots were excised, digested with trypsin (Promega, Madison, WI), mixed with α-cyano-4-hydroxycinnamic acid (CHCA; Sigma-Aldrich) in 50% acetonitrile/0.1% trifluoroacetic acid, and used for MALDI-TOF analysis (Microflex LRF 20, Bruker Scientific LLC, Billerica, MA, USA) as described by Fernandez et al. [45]. Spectra were collected from 300 shots per spectrum over an *m*/*z* range of 600–3000 and calibrated using a two-point internal calibration of trypsin autodigested peaks (*m*/*z* 842.5099, 2211.1046). The peak list was generated using Flex Analysis 3.0. The thresholds used for peak-picking were 500 for the minimum resolution of monoisotopic mass and 5 for S/N. The search program MASCOT, developed by Matrixscience (http://www.matrixscience.com), was used, and the MASCOT probability-based molecular weight search (MOWSE) score was calculated for PMF. For database search, the following parameters were used: trypsin included as the cleaving enzyme, a maximum of one missed cleavage, iodoacetamide (Cys) as a complete modification, oxidation (Met) as a partial modification, monoisotopic masses, and a mass tolerance of ±0.1 Da. The PMF acceptance criteria were based on probability scoring as follows: −10∗Log (P), where P is the probability that an observed match is a random event, and a score greater than 68 is considered significant (*p* < 0.05).

### 4.13. Antibody Blocking Assay by Neutralizing Antibodies

To find causative factors associated with cytotoxicity of NKL-derived EVs against cancer, neutralizing antibodies of β-actin (PA1-46296, Thermo Fisher), fibrinogen (ab34269, Abcam, Cambridge, UK), and isotype control (rabbit IgG, polyclonal; Abcam) were used. NKL-derived EVs (20 μg/well in 96-well plate) mixed with individual neutralizing antibodies (500 ng/mL) were incubated for 2 h on ice, and the mixture was applied to 5 different cancer cell lines for viability and cytotoxicity assay.

### 4.14. Recombinant β-Actin and Fibrinogen Treatment

To verify the role of β-actin and fibrinogen, 5 different cancer cell line cells were treated with purified recombinant β-actin (APHL99-A, Cytoskelecton, Denver, CO, USA) and fibrinogen (ab62394, Abcam), and their effect on cancer cells was evaluated using LDH assay.

### 4.15. Statistical Analysis

All data were compared via one-way analysis of variance (ANOVA) using the Statistical Package for the Social Sciences (SPSS, version 14.0K) program. The data are expressed as means ± standard error of measurement (SEM). Group means were considered significantly different at *p* < 0.05, as determined by the protected least-significant difference (LSD) test when ANOVA indicated an overall significant treatment effect (*p* < 0.05).

## 5. Conclusions

In the present study, the anticancer potential of isolated EVs from expanded and activated NKLs was evaluated, and our data identified a number of molecules, such as Fas ligand, TRAIL, NKG2D, β-actin, and fibrinogen, as effector candidates based on the proteomic analysis and functional study. The results of the present study suggest the possibility of NK-cell-derived EVs as a viable immunotherapeutic strategy for cancer.

## Figures and Tables

**Table 1:** List of identified proteins in isolated EVs by peptide mass fingerprinting (PMF) analysis.

Spot ID	Protein Name	Gene Name	Calculated pI	Normimal Mass (Mr) ^1)^	Sequence Coverage (%)	Score ^2)^	Protein Intensity (% vol) ^3)^		Fold Change (NKLs/CON)	*p* Value ^4)^
							CON	NKLs		
2897	Fibrinogen gamma chain, isoform CRA_o	FGG	5.54	47971	33	109	0.03	1.16	43.94	0.021
	Fibrinogen gamma chain, isoform CRA_j		6.02	48277	33	109				
2886	Fibrinogen beta chain, isoform CRA_g		8.63	50940	18	84	0.12	4.68	39.32	0.001
	Fibrinogen beta chain, isoform CRA_d		8.33	52759	18	84				
2876	Fibrinogen beta chain, isoform CRA_d		8.33	52759	24	98	0.01	0.27	21.26	0.022
2889	Fibrinogen beta chain, isoform CRA_g		8.63	50940	28	96	0.08	1.21	16.03	0.011
2888					22	84	0.14	0.44	3.02	0.049
2896	ACTB protein, partial	ACTB	5.55	40536	34	81	0.10	1.38	13.72	0.035
2885					33	107	0.08	0.86	11.35	0.016
2898					36	98	0.07	0.63	8.72	0.004
2901					39	142	0.17	1.43	8.35	0.001
2893					43	199	0.20	0.88	4.42	0.006
2884					37	109	0.53	1.57	2.99	0.062
2882					50	142	0.42	1.04	2.47	0.002
2814					28	77	0.68	1.31	1.94	0.005
2879					46	136	2.02	3.34	1.66	0.027
2620					28	98	0.29	0.44	1.54	0.003
2865					41	108	0.34	0.50	1.47	0.049
2878					48	170	13.68	15.87	1.16	0.040
2872	L-plastin polypeptide	LCP1	5.41	64352	20	100	0.03	0.35	12.72	0.027
2906	Tyrosine 3-monooxygenase/tryptophan 5-monooxygenase activation protein zeta polypeptide	YWHAZ	4.73	27867	35	116	0.10	1.27	12.23	0.052
2910					31	89	0.05	0.45	8.46	0.023
2908	Tyrosine 3-monooxygenase/tryptophan 5-monooxygenase activation protein epsilon	YWHAE	4.63	29326	27	68	0.06	0.67	10.37	0.016
2877	PA2G4 protein, partial	PA2G4	9.08	45579	18	105	0.03	0.32	9.94	0.005
2899	Cytokeratin 9	KRT9	5.19	62320	30	85	0.09	0.87	9.77	0.003
2907	Proteasome (prosome, macropain) subunit, alpha type, 5	PSMA5	4.74	26579	46	139	0.09	0.65	7.23	0.026
2874	Human rab GDI	GDI2	5.94	51088	40	189	0.11	0.72	6.81	0.002
2858	Heat shock protein HSP 90-beta precursor, partial	HSP90AB1	4.73	90309	27	141	0.14	0.69	5.03	0.001
2862	Heat shock protein HSP 90-beta isoform c	HSP90AB1	4.98	82611	29	135	0.41	1.34	3.27	0.034
2902	Annexin A5	ANXA5	4.94	35971	62	256	0.12	0.58	4.78	0.015
2891	Glutathione S-transferase	GSTM4	5.43	23595	61	113	0.10	0.47	4.44	0.026
2883	L-lactate dehydrogenase B chain isoform LDHB	LDHB	5.71	36900	32	142	0.31	1.15	3.75	0.006
2843	Gamma-actin, partial	ACTG1	5.65	26147	52	147	1.04	2.97	2.86	0.014
2873	Protein disulfide isomerase family A, member 3, isoform CRA_b	PDIA3	6.42	55328	42	230	0.41	1.04	2.53	0.004
	Protein disulfide isomerase family A, member 3, isoform CRA_a	PDIA3	6.78	54454	42	230				
2894	Apolipoprotein E	APOE	5.81	36242	38	104	0.43	0.99	2.29	0.049
2870	T-complex protein 1 subunit epsilon isoform d	CCT5	5.86	49951	38	126	0.34	0.72	2.10	0.017
2871	T-complex protein 1 subunit theta isoform 2	CCT8	5.25	58179	29	176	0.28	0.52	1.84	0.029
2645	Valosin-containing protein	VCP	5.19	89972	30	172	0.25	0.46	1.80	0.046
2881	Apolipoprotein A-IV	APOA4	5.23	45307	40	147	0.26	0.42	1.64	0.046

^1)^ The nominal mass is the integer mass of the most abundant naturally occurring stable isotope of an element. The nominal mass of a molecule is the sum of the nominal masses of the elements in its empirical formula. ^2)^ MASCOT probability-based MOWSE (molecular weight search) score calculated for PMF. Protein score is −10∗Log(P), where P is the probability that the observed match is a random event and greater than 66 are significant (*p* < 0.05). ^3)^ Protein intensity indicated average of controls and 5 individuals. ^4)^ Statistical significance between control EVs (CON) and NKLs-derived EVs (NKLs) was determined by a *t*-test.

## Supplementary Materials

The following are available online. Figure S1: Proteins increased in NKL-derived EVs. Figure S2: Examination of anticancer potential of NKL-derived proteins using neutralizing antibodies.
