# Supplementary material for: Proteome Analysis of Human Natural Killer Cell Derived Extracellular Vesicles for Identification of Anticancer Effectors
Source: Molecules. 2020 Nov 9;25(21):5216. doi: 10.3390/molecules25215216 (PMC7664935; doi:10.3390/molecules25215216)
Supplement: Supplementary file 1 [file molecules-25-05216-s001.pdf]

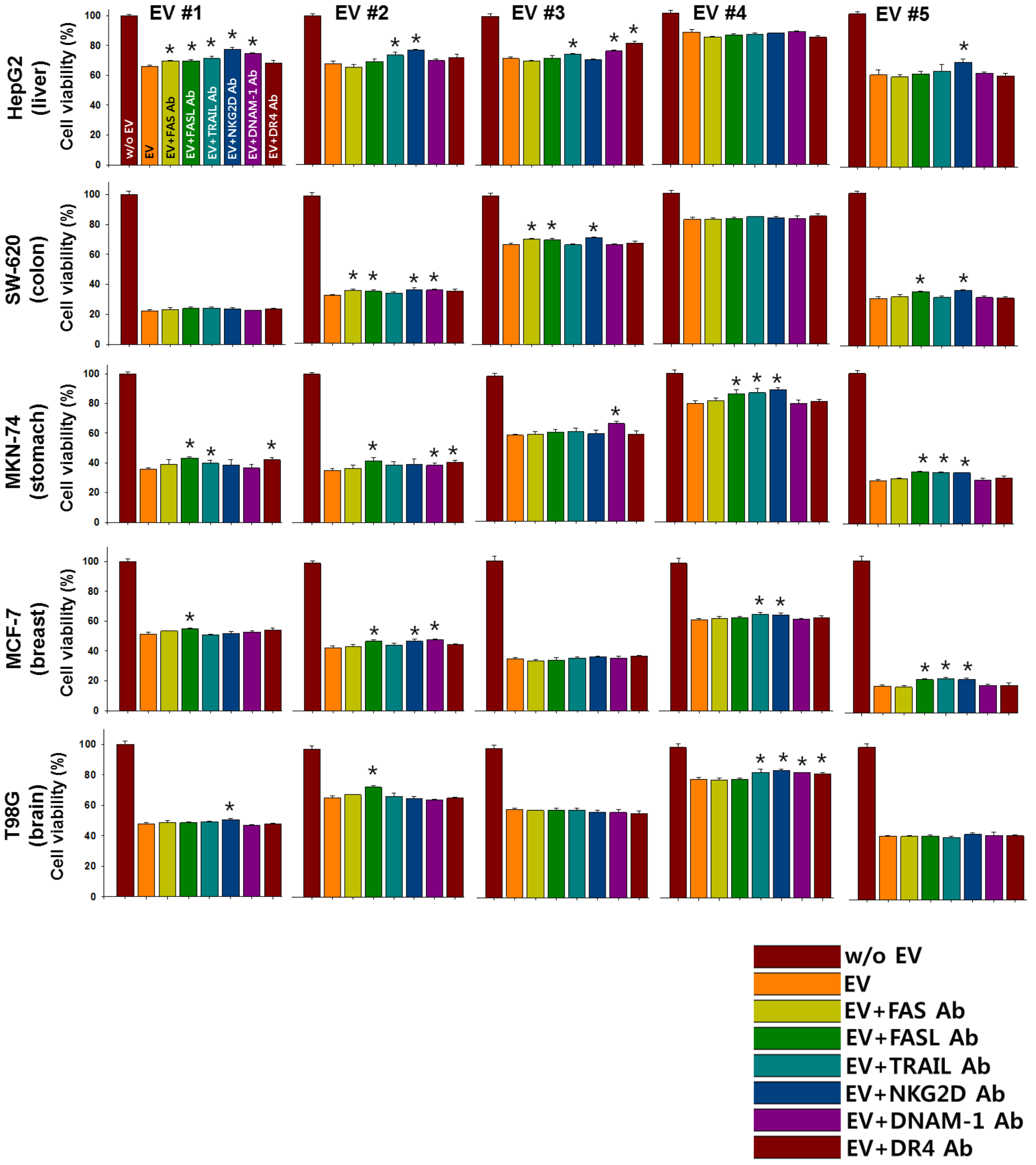

Fig. S1 Examination of anti-cancer potential of NKLs-derived proteins using neutralizing antibodies. Individual EVs mixed with/without neutralizing antibodies were applied to 5 different types of cancer cells, and cytotoxicity and cell viability of cancer cells were measured. Experiments were performed in triplicates. Significant differences were determined via ANOVA, with p values indicated as $*p<0.05$ compared to the EVs without neutralizing antibodies.
